# Congenital thymoma

**DOI:** 10.4322/acr.2021.327

**Published:** 2021-09-22

**Authors:** Momal Tara Chand, Jacob Edens, Tayson Taixin Lin, David Goshorn, Truc Pham

**Affiliations:** 1 Ascension St John Hospital, Department of Pathology, Detroit, Michigan, USA; 2 Sacred Heart Children’s Hospital, Spokane, Washington, USA

**Keywords:** Congenital Abnormalities, Hospitals, Pediatric, Thymoma, Thymus Hyperplasia, Thymus Neoplasms

## Abstract

Thymomas are a heterogeneous group of tumors arising from the epithelium of the thymus. They are categorized by the proportion of neoplastic epithelia to lymphocytes and by the degree of cytologic atypia. Thymomas constitute 0.2-1.5% of all malignancies and nearly all occur in patients over 20 years. We reviewed the available literature and found less than 50 cases of thymoma reported in children (<18 years of age), the youngest being 4 years old, and no cases in newborns. They represent less than 1% of all mediastinal tumors in children. Due to the limited number of cases in the pediatric population, the diagnosis and treatment in this population is extremely challenging. Thymomas in all age groups may be associated with paraneoplastic syndromes, being myasthenia gravis the most common, which is associated with a worse prognosis in the pediatric population. We present the first case of a newborn infant with congenital thymoma. This case demonstrates a rare tumor in an unusual age group and emphasizes the importance of multidisciplinary teamwork in the decision-making and management of this condition.

## INTRODUCTION

Thymomas are neoplasms of the thymic epithelium characterized by admixture of epithelial cells and mature lymphocytes. They usually develop in the fifth to seventh decade of life^1^ but are exceedingly rare in children, with only a handful of reports documented in literature.^2^ We carried out a comprehensive literature search on PubMed and Google Scholar using the key words “Congenital” and “thymoma”, and found no cases reporting thymomas in newborns. To the best of our knowledge, less than 50 cases of thymomas are described in children less than 18 years of age, the youngest case in the literature was reported by the European Cooperative Study Group for Pediatric Rare Tumors (EXPeRT) database. They did a retrospective analysis of pediatric patients less than 18 years with thymic tumors over the span of 12 years (2000-2012) and identified only 36 patients with thymic neoplasms across 4 European countries including France, Germany, Italy, and Poland. Of the 36 cases reported, 16 children were identified with thymoma, median age 11 years (range 4 to 17 years) and 20 with thymic carcinoma, median age 14 (range 4.5-19 years).^3^ Another study from Italy on rare tumors of pediatric age group, Tumori Rari in Età Pediatrica [Rare Tumors in Pediatric Age] (TERP), reported only 9 patients with thymic neoplasm (4 with thymoma and 5 with thymic carcinoma) over the span of 9 years (2000-2009).^4^ Even one of the largest studies carried out by Thymic International Group which described 1,470 cases of thymomas, spanning across 11 countries, had no patients below 12 years of age.^5^ Herein, we report the first case of congenital thymoma in a male infant.

## CASE REPORT

**Figure 1:**
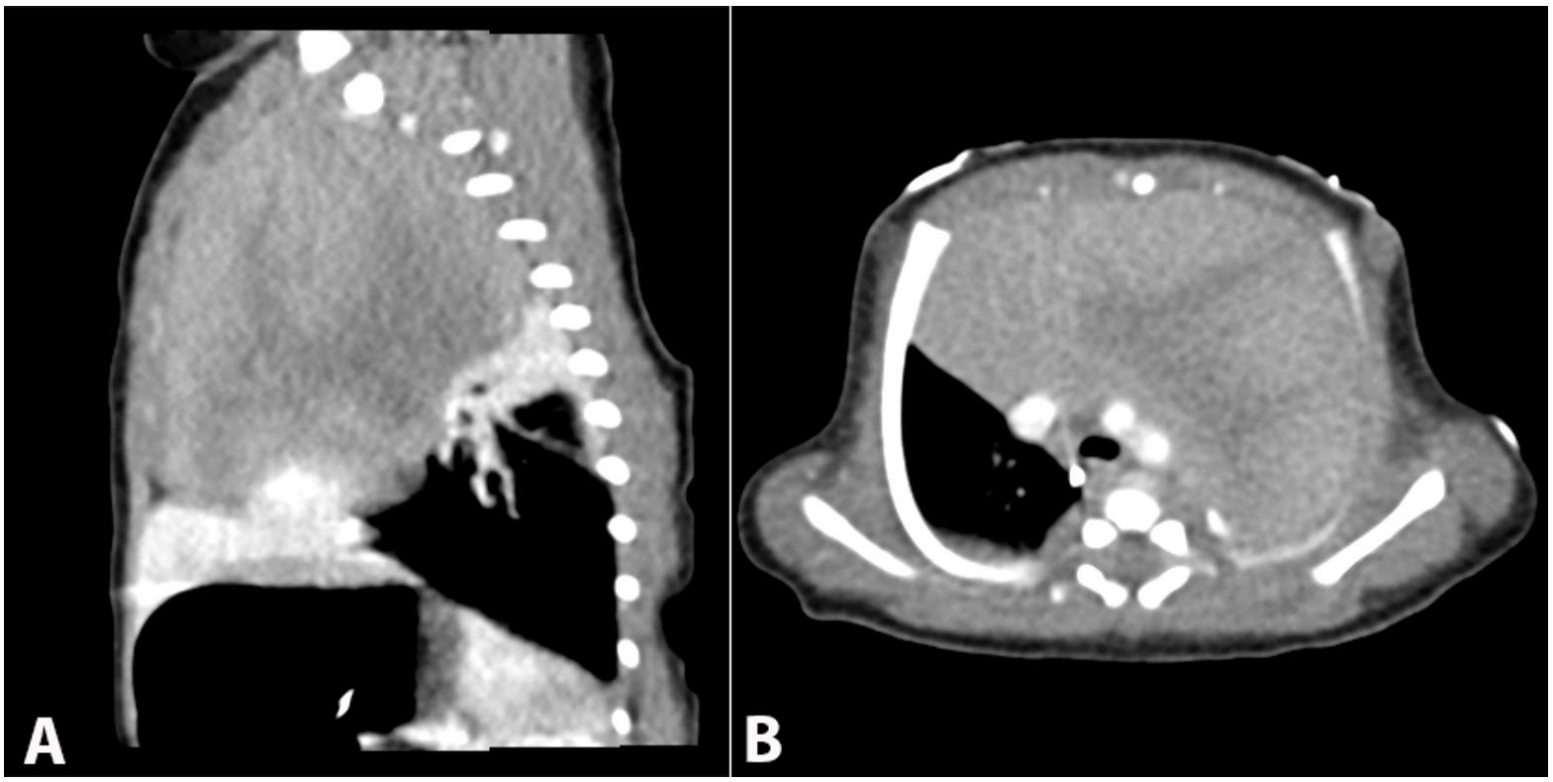
**A** – Sagittal contrast-enhanced thoracic computed tomography (CT) demonstrates a large, heterogeneously enhancing, anterior mediastinal mass. No calcification or necrosis is noted. The mass causes passive atelectasis of the adjacent right middle and upper lung lobes; **B** – Axial contrast-enhanced thoracic CT demonstrates a large anterior mediastinal mass with heterogeneous enhancement. The lesion exerts mass effect on the great vessels, displacing them posteriorly. Extrinsic compression on the tracheal is also noted, but the tracheal remains patent. No area of necrosis, calcification, or lymphadenopathy noted.

The patient is a male infant born at 39-week gestation with an unremarkable prenatal history via uncomplicated vaginal delivery. Normal anatomy was reported at a 23-week ultrasound examination. At birth, APGAR scores were 6/7/7 when the patient was diagnosed with transient tachypnea of the newborn. A chest X-Ray revealed a large mediastinal mass. Computed tomography revealed a 9x5x5 cm solid, heterogeneous mass in the anterior mediastinum, which displaced the heart, great vessels and lungs without invasion or narrowing of the airway (Figure 1). At this time the differential diagnosis included: thymic hyperplasia, thymoma, lymphoblastic lymphoma, germ cell tumor, teratoma and hiatal hernia.

Ultrasound guided biopsy was performed and submitted for microscopic, flow cytometric and molecular diagnostic analysis. Histologic examination demonstrated a proliferation of small ovoid lymphocytes in the background of blood vessels and larger epithelial cells. Immunohistochemical staining of the biopsy revealed a majority population of lymphocytes that were immunoreactive with CD1a, CD3, and Terminal deoxynucleotidyl transferase (TdT) and non-reactive with CD34 with a background of pankeratin reactive epithelial cells. There was also variable CD10 reactivity amongst lymphocytes and a small number of lymphocytes reactive with CD20. No malignant features of thymic carcinoma were identified. Flow cytometry revealed an 81% population of CD4/8 (++)/CD2/CD3(var)/CD5/CD7(+), CD20/CD34(-) lymphocytes. There was no increased population of blasts and no clonal B-cell population detected. Molecular diagnostics detected no monoclonal T-Cell receptor γ, which ruled out T-lymphoblastic lymphoma. Interpretation of these results narrowed the differential to: Thymic hyperplasia vs. thymoma vs. normal thymus due to simple variation.

**Figure 2:**
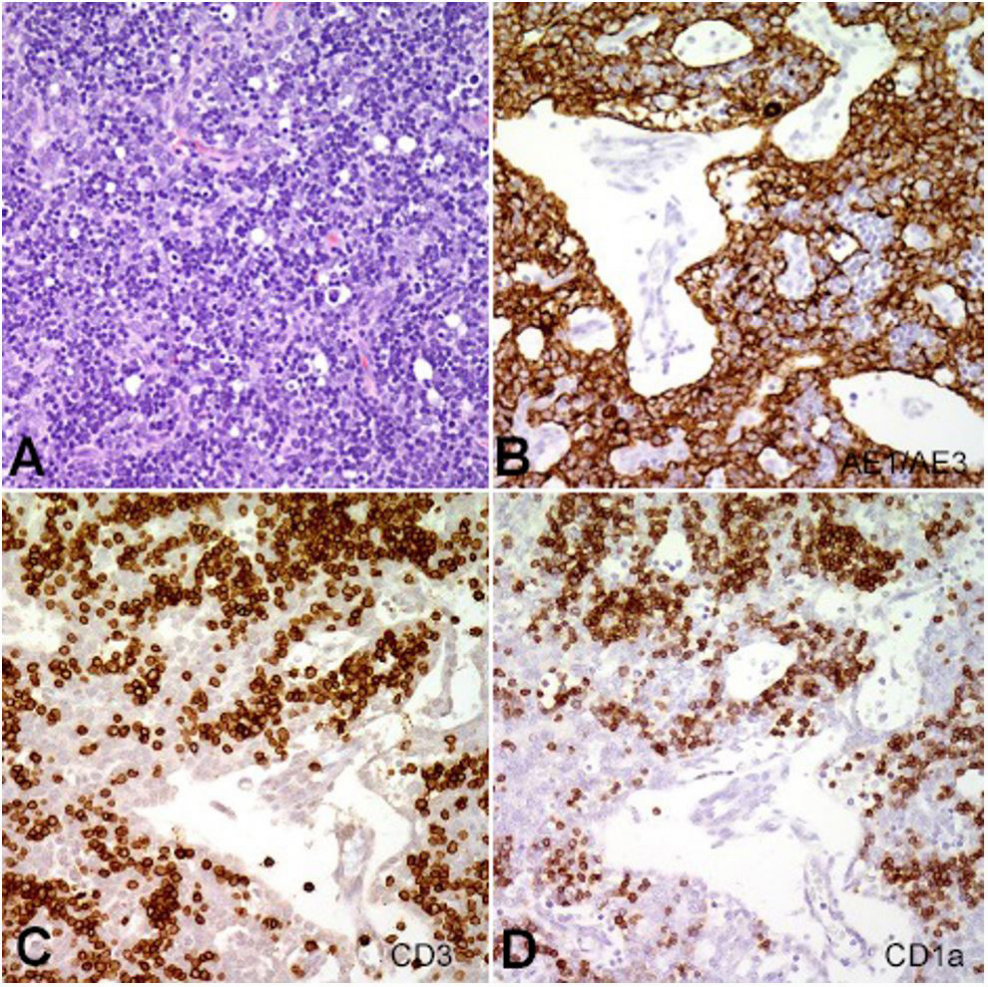
**A** – Hematoxylin eosin-stained section reveals mixed population of pleomorphic thymocytes in a background of bland appearing lymphocytes. Immunohistochemical stain reveals large proliferation of (**B**) CK AE1/AE3 positive thymocytes, and (**C** and **D**) CD3 and CD1a positive thymic lymphocytes.

At this point, the case was discussed at a multidisciplinary tumor board. The clinical team favored a diagnosis of germ cell tumor because of the relative commonality and evidence of tumor heterogeneity on imaging. The use of preoperative steroids was discussed in an attempt to shrink the mass but was decided against in order not to delay surgery and to avoid the risk of complicated wound healing. The decision to proceed with surgery was made and median sternotomy was performed with complete resection of the mass. Gross examination revealed a 162 gms, 12.5 x 8.0 x 3.5 cm portion of encapsulated lobulated tissue resembling normal thymus without evidence of fibrosis or calcification. The cut surface was tan-pink, lobulated and soft with focal hemorrhage measuring up to 0.8 cm. A frozen section analysis at the time of the procedure revealed a proliferation of cells consistent with thymoma or thymic hyperplasia. Microscopic examination of permanent sections revealed loss of normal thymic architecture with a mixed B1/B2 thymoma pattern (Figure 2).

The B1 portion was represented by abundant thymocytes with inconspicuous epithelial components and foci of abortive medullary differentiation. The B2 portion was represented by epithelial cells palisading around open perivascular spaces containing lymphocytes (Figure 2B). The epithelial cells were highlighted by immunohistochemical stain cytokeratin AE1/AE3 (Figure 2B). No evidence of germ cell tumor or teratoma was detected. Diagnosis of thymoma was rendered. The tumor had a pathologic stage of pT1 pNX and was a stage I according to modified Masaoka staging system. Inquiry into the patient’s family history revealed no known history of thymoma or myasthenia gravis. The patient had an uneventful postoperative period and recovered without incident. Four years later the patient has developed normally with a functional immune system. The clinicians decided to delay the immunization schedule in order to give the patient’s T-Cells a chance to develop.

## DISCUSSION

The management of rare neoplasms and cancers pose significant diagnostic challenges, sometimes with major consequences for patients' quality of life and outcome. Thymic epithelial neoplasm (TETs) are an excellent example of rare tumors which can pose a significant diagnostic and therapeutic dilemma even for an experienced pathologist. They are uncommon neoplasms arising from the thymic epithelium accounting for less than 1% of all mediastinal tumors in the pediatric age group. Due to the limited number of cases in the pediatric population, the diagnosis and treatment in this population is extremely challenging and almost all the information for pediatric cases come from the adult cases.

Like adults, the clinical manifestations in children vary from incidentally detected asymptomatic masses to being symptomatic with dysphagia, chest pain and respiratory distress secondary to thoracic mass effect.^3^^,^^6^ Stage III and Stage IV thymomas may also present as superior vena cava syndrome.^7^ They are generally encapsulated and non-invasive; however, integrity of the capsule is a significant determinant of prognosis. This family of tumors is also commonly associated with paraneoplastic syndromes, such as myasthenia gravis, pure red cell aplasia, hypogammaglobulinemia and Sjögren syndrome manifesting more commonly in adults rather than in children.^3^^,^^6^ Other clinical manifestations include gastrointestinal disorders (chronic ulcerative colitis), collagen and autoimmune disorders (Sjogren’s syndrome, scleroderma and polymyositis), dermatologic disorders (pemphigus, alopecia), endocrine disorders (Cushing’s syndrome), renal disease (nephrosis) and hematologic syndromes (red cell aplasia, agranulocytosis).^3^^,^^6^^,^^8^ Myasthenia gravis is the most common paraneoplastic syndrome in children especially in children <10 years.^6^^,^^9^ Its presence is also correlated with poor prognosis.^6^^,^^9^

The histologic classification of thymomas has taken a center stage. Various groups have developed classifications for thymic epithelial neoplasm over the years, including the World Health Organization (WHO). However, the Masaoka-Koga^10^ classification remains the most common and frequently applied clinical staging system and is an excellent predictor of the prognosis of thymoma. The most recent WHO classification, revised in 2015, divides thymic neoplasms into types A, AB, B1, B2, B3; reflecting a spectrum of histopathological changes. Thymoma types A, AB, and B1 have relatively good outcomes; B2 and B3 are more aggressive with intermediate survival rates; and thymic carcinomas have the worst outcome.^3^^,^^4^ Histologically, the tumors in pediatric age group are the same as adults, however, very little is known about its outcome and prognosis in this pediatric age group and nearly all experience is derived from the adult groups. The European Cooperative Study Group for Pediatric Rare Tumors (EXPeRT) (including 36 patients) and The Tumori Rari in Eta Pediatrica (Rare Tumors in Pediatric Age) (TREP) project (including 9 patients) confirmed that the pediatric thymic tumors behave the same as adults. Liang et al.^11^ reviewed pediatric cases of thymoma of over 30 years and found that patients younger than 10 are predominantly male with advanced stage and those older than 10 are predominantly females with better disease outcome. He also argued, in his paper, that the type of paraneoplastic condition associated with thymomas also have a prognostic significance along with the stage and histologic subtype.

Treatment and prognosis are based on the Masaoka Staging System,^10^ which evaluates for microscopic invasion and distal metastases. Because most are well encapsulated, surgical excision is usually curative. Neoadjuvant chemotherapy and/or postoperative chemoradiotherapy are offered in cases of borderline surgically resectable tumors. For patients with unresectable disease or extensive medical comorbidities, systemic therapy or radiotherapy is indicated.

## CONCLUSION

Congenital thymoma is a nearly unheard-of condition. Our case represents the first confirmed congenital thymoma, which highlights some of the challenges, and this displays the need for a multidisciplinary team to manage these patients. This case reminds us that even unheard-of pathologies can and do exist, and an open mind is sometimes required to reach the true diagnosis.
